# Supplementary material for: Comparison of Nutritional Profiles of Super Worm (Zophobas morio) and Yellow Mealworm (Tenebrio molitor) as Alternative Feeds Used in Animal Husbandry: Is Super Worm Superior?
Source: Animals (Basel). 2022 May 17;12(10):1277. doi: 10.3390/ani12101277 (PMC9137835; doi:10.3390/ani12101277)
Supplement: Supplementary file 1 [file animals-12-01277-s001.zip › animals-1706660-supplementary.pdf]

Table S1. Proximate composition of full-fat larval meal.

Insect species	Substrate	Time of harvest	Yield (g)	Proteins (%) (n = 3)	Crude fat (%) (n = 3)	Crude ash (%) (n = 3)	Crude fibre (%) (n = 3)	Protein digestibility (%) (n=3)
**TM**	M	1	221.20	37.27	31.87	5.02	6.17	84.93
**TM**	K	1	178.41	40.87	28.46	5.73	7.34	84.67
**TM**	S	1	206.57	37.96	31.03	6.84	8.51	85.59
**TM**	M	2	291.16	37.30	33.54	4.80	8.36	83.05
**TM**	K	2	273.38	41.90	30.23	4.79	5.28	84.30
**TM**	S	2	226.90	42.34	28.85	5.13	8.12	84.66
**TM**	M	3	302.40	39.63	33.53	4.91	6.72	85.06
**TM**	K	3	323.68	42.11	27.16	5.27	6.33	84.80
**TM**	S	3	226.90	42.14	24.83	5.16	5.53	83.32
**ZM**	M	1	130.00	36.02	33.69	3.76	5.95	83.77
**ZM**	K	1	166.12	41.36	33.13	2.89	5.73	84.05
**ZM**	S	1	167.08	33.94	33.41	2.99	5.89	82.05
**ZM**	M	2	132.03	42.26	37.58	2.88	6.72	83.92
**ZM**	K	2	177.02	42.82	44.48	3.21	7.96	82.23
**ZM**	S	2	136.74	42.81	36.50	3.22	7.00	83.18
**ZM**	M	3	139.83	39.59	32.35	2.69	9.12	83.61
**ZM**	K	3	177.03	37.48	34.47	3.00	7.70	83.56
**ZM**	S	3	111.88	35.95	40.81	3.13	8.91	83.45

Results are presented as mean value on dry matter. TM. *Tenebrio molitor*; ZM, *Zophobas morio;* M. substrate that included cabbage, carrot and flaxseed; K. substrate that included cabbage; S, substrate that included carrot; times 1, 2 and 3 represent 90, 97 and 104 days, respectively. Protein content is expressed by using nitrogen-to-protein conversion factor of kp 4.76.

Table S2. Amino acid composition of full-fat larval meal.

Insect species	Substrate	Time of harvest	Asp	Tyr	Arg	Ser	Glu	Pro	Gly	Ala	Cys	Val	Met	Isl	Leu	Thr	Phe	His	Lys
			Non-essential									Essential							
			% (n = 3)																
**TM**	M	1	8.98	9.68	6.81	3.97	12.39	10.11	4.88	6.29	1.46	5.92	1.85	5.16	8.02	4.57	5.00	4.18	6.67
**TM**	K	1	8.47	9.64	6.87	3.86	11.24	10.73	4.45	5.62	1.27	5.44	1.92	5.04	7.46	3.62	4.63	4.33	6.51
**TM**	S	1	8.59	10.08	7.05	3.99	11.37	11.27	4.48	4.46	1.33	5.46	2.04	5.29	7.25	3.99	4.40	4.67	6.72
**TM**	M	2	9.74	9.66	7.10	4.18	13.09	10.49	5.23	5.33	1.50	6.14	1.79	5.29	8.19	3.73	4.33	4.37	6.99
**TM**	K	2	8.67	9.15	6.07	3.70	11.41	9.38	4.48	4.45	1.37	5.23	1.75	4.74	7.18	4.37	4.77	3.99	7.17
**TM**	S	2	9.02	9.41	6.74	3.95	12.04	10.45	4.83	4.87	1.51	5.61	1.79	5.06	7.55	4.29	4.96	4.25	6.62
**TM**	M	3	8.26	8.90	5.96	3.62	11.21	9.16	4.52	4.58	1.42	5.34	1.65	4.61	7.29	4.02	4.36	3.85	6.26
**TM**	K	3	8.22	8.76	6.21	3.66	10.78	9.52	4.23	4.27	1.29	5.00	1.63	4.60	6.75	4.21	4.20	4.00	6.08
**TM**	S	3	8.57	8.77	6.13	3.72	11.33	9.44	4.48	4.53	1.43	5.36	1.67	4.70	7.31	4.06	4.75	3.87	6.25
**ZM**	M	1	9.34	12.12	8.14	4.83	13.51	10.60	5.44	6.68	0.00	7.02	1.92	6.88	7.29	5.09	4.90	5.16	7.22
**ZM**	K	1	8.15	10.95	7.37	4.16	11.58	9.36	4.85	6.11	0.00	6.33	1.77	5.99	6.67	4.50	4.11	4.62	6.28
**ZM**	S	1	9.48	11.38	7.85	4.71	13.87	10.07	5.62	7.26	0.00	7.20	2.02	6.74	7.46	4.96	5.29	4.78	7.13
**ZM**	M	2	8.11	10.60	7.56	4.19	11.65	9.11	4.63	5.79	0.00	6.07	1.88	6.07	6.84	4.53	4.03	4.74	6.41
**ZM**	K	2	9.31	11.07	8.18	4.77	13.37	9.89	5.36	6.83	0.00	6.95	1.65	6.47	7.25	5.00	4.83	5.07	6.95
**ZM**	S	2	7.92	10.44	7.02	4.04	11.17	8.50	4.56	5.76	0.00	6.03	1.73	5.71	6.39	4.35	4.19	4.45	6.08
**ZM**	M	3	9.20	11.58	7.30	4.87	13.62	10.27	5.72	7.43	0.00	7.37	1.44	6.45	7.23	4.81	5.20	4.87	6.64
**ZM**	K	3	9.90	11.99	8.53	4.88	14.40	10.03	5.67	7.23	0.00	7.23	2.02	6.97	7.68	5.15	5.41	4.95	7.43
**ZM**	S	3	9.38	11.69	8.15	4.88	13.68	10.35	5.66	7.33	0.00	7.20	2.00	6.68	7.46	4.95	5.27	4.88	7.20

Results are presented as mean value, as % AA of protein content (kp 4.76). TM, *Tenebrio molitor*; ZM, *Zophobas morio;* M, substrate that included cabbage, carrot and flaxseed; K, substrate that included cabbage; S, substrate that included carrot; times 1, 2 and 3 represent 90, 97 and 104 days, respectively; Asp, aspartic acid ; Thr, threonine; Ser, serine; Glu, glutamic acid; Pro, proline; Gly, glycine; Ala, alanine; Cys, cystine; Val, valine; Met, methionine; Isl, isoleucine; Leu, leucine; Tyr, tyrosine; Phe, phenylalanine; His, histidine; Lys, lysine; Arg, arginine.

Table S3. Mineral content of full-fat larval meal.

Insect species	Substrate	Time of harvest	Ca	K	Mg	Na	Fe	Zn	Mn	Cu	P
						mg/100g (n = 3)					
**TM**	M	1	24.30	827.42	181.19	168.72	5.05	12.04	0.82	1.51	753.23
**TM**	K	1	26.82	878.14	192.70	117.72	4.22	13.59	1.30	1.89	794.90
**TM**	S	1	25.95	741.74	205.82	148.67	4.16	12.48	1.07	1.83	800.28
**TM**	M	2	26.60	862.89	216.83	153.61	4.27	11.42	0.88	2.02	754.81
**TM**	K	2	32.28	762.27	227.71	153.27	5.20	13.76	1.14	2.35	782.99
**TM**	S	2	27.28	863.09	267.77	160.52	5.88	15.60	1.63	2.75	888.77
**TM**	M	3	29.54	868.59	240.66	144.86	5.39	11.04	0.97	1.84	751.56
**TM**	K	3	29.02	831.48	238.46	157.12	6.66	13.11	1.23	2.29	833.68
**TM**	S	3	24.89	786.33	277.14	181.96	5.38	16.05	1.95	2.37	915.80
**ZM**	M	1	38.68	601.40	98.12	155.86	8.91	7.48	0.85	1.04	547.11
**ZM**	K	1	59.54	590.27	91.16	138.51	6.24	5.69	0.97	1.27	628.47
**ZM**	S	1	39.56	493.19	71.95	108.00	6.02	5.31	1.03	0.94	541.14
**ZM**	M	2	66.85	615.97	95.52	183.47	11.58	7.49	0.97	1.20	570.06
**ZM**	K	2	64.25	665.71	100.23	152.55	4.29	7.26	1.13	1.31	566.04
**ZM**	S	2	40.74	643.96	100.98	167.23	6.65	7.03	1.19	1.21	605.15
**ZM**	M	3	65.21	615.27	90.68	158.66	3.98	6.84	0.98	1.11	651.15
**ZM**	K	3	55.92	552.36	99.87	187.70	10.77	6.41	1.20	1.32	625.99
**ZM**	S	3	65.81	641.58	95.16	154.43	4.20	6.70	0.94	1.05	612.89

Results are presented as mean value, as mg/100g of dry matter. TM, *Tenebrio molitor*; ZM, *Zophobas morio;* M, substrate that included cabbage, carrot and flaxseed; K, substrate that included cabbage; S, substrate that included carrot; times 1, 2 and 3 represent 90, 97 and 104 days, respectively.

Table S4. Fatty acid profile of full-fat larval meal.

Insect species	Substrate	Time of harvest	C8:0	C10:0	C12:0	C14:0	C15:0	C16:0	C16:1	C17:0	C17:1	C18:0	C18:1n9c	C18:2n6c	C20:0	C18:3n3	SFA	MUFA	PUFA	n6/n3
			% fatty acids (n = 3)																	
**TM**	M	1	0	0	0.2	2.0	0.1	16.2	0.6	1.4	0	3.0	40.3	25.6	0	10.5	22.94	40.96	36.1	2.5
**TM**	K	1	0	0	0.3	2.1	0.1	18.8	0.8	1.6	0	2.9	46.7	25.4	0	1.4	25.7	47.42	26.8	18.1
**TM**	S	1	0	0	0.3	2.8	0.1	18.2	0.9	1.7	0	3.1	47.7	24.2	0	0.9	26.19	48.65	25.16	26.9
**TM**	M	2	0	0	0.2	2.1	0.1	15.5	0.6	1.6	0	2.5	41	23.6	0	12.9	21.88	41.56	36.56	1.8
**TM**	K	2	0	0	0.3	2.2	0.1	18.2	0.7	1.8	0	2.3	48.7	24.5	0	1.2	24.89	49.39	25.72	20.4
**TM**	S	2	0	0	0.3	2.5	0.1	18.6	0.8	1.9	0	2.6	47.1	24.8	0	1.1	26.1	47.99	25.91	22.5
**TM**	M	3	0	0	0.2	2.2	0.1	15.3	0.7	1.5	0	2.5	40.6	23	0	13.9	21.83	41.3	36.86	1.6
**TM**	K	3	0	0	0.3	2.4	0.2	18.3	0.7	2.0	0	2.9	46.8	25.4	0	1.1	26.03	47.5	26.47	23.1
**TM**	S	3	0	0	0.3	2.7	0.2	18.9	0.9	2.0	0	2.2	48.6	23.1	0	1.0	26.32	49.58	24.1	23.1
**ZM**	M	1	0.6	0.1	0.1	1.2	0.3	26.0	2.8	0.7	0.5	5.8	30.6	18.1	0.2	13.1	34.9	33.9	31.3	1.4
**ZM**	K	1	0.5	0.1	0.1	1.2	0.3	30.4	2.4	0.9	0.5	7.7	28.7	25.5	0.2	1.5	41.4	31.6	27.0	16.7
**ZM**	S	1	0.6	0.1	0.1	1.3	0.3	31.6	2.4	0.8	0.5	7.3	29.0	24.6	0.2	1.1	42.3	32.0	25.7	21.4
**ZM**	M	2	0.7	0.1	0.1	1.2	0.3	26.1	3.0	0.7	0.5	5.7	30.6	18.1	0.2	12.9	35.0	34.1	30.9	1.4
**ZM**	K	2	0.6	0.1	0.1	1.3	0.3	31.0	2.7	0.9	0.6	7.1	28.7	24.9	0.2	1.5	41.7	31.9	26.4	16.7
**ZM**	S	2	0.6	0.1	0.1	1.4	0.3	32.8	2.6	0.9	0.5	6.9	29.4	23.1	0.2	1.1	43.2	32.5	24.2	21.1
**ZM**	M	3	0.6	0.1	0.1	1.3	0.3	26.6	3.0	0.8	0.5	5.8	30.7	18.2	0.2	11.8	35.8	34.2	30.0	1.5
**ZM**	K	3	0.7	0.1	0.1	1.4	0.4	31.1	3.0	1.0	0.7	6.5	28.6	23.9	0.2	2.3	41.4	32.3	26.2	10.3
**ZM**	S	3	0.7	0.1	0.1	1.4	0.3	32.6	0.8	0.9	0.5	7.2	29.6	24.3	0.2	1.3	43.4	30.9	25.6	18.7

Results are presented as mean value, as% fatty acids in total identified FA. TM, *Tenebrio molitor*; ZM, *Zophobas morio;* M, substrate that included cabbage, carrot and flaxseed; K, substrate that included cabbage; S, substrate that included carrot; times 1, 2 and 3 represent 90, 97 and 104 days of harvest, respectively; SFA, saturated fatty acid; MUFA, monounsaturated fatty acid; PUFA, polyunsaturated fatty acid.

Table S5. Multi-objective optimization of the outputs of the ANN.

**Proximate composition**		**Amino acid (% on protein content)**		**Mineral content (mg/100g d.m.)**		**Fatty acid composition (% in total FA content)**	
	(g)	Threonine	4.96	Ca	24.89	C18:2n6c	25.6
Yield	302.40	Valine	7.13	K	786.33	C18:3n3	10.5
	(% d.m)	Methionine	2.02	Mg	277.14	n6/n3	2.44
Crude protein	39.63	Isoleucine	6.72	Na	181.96		
Crude fat	33.53	Leucine	7.48	P	915.80		
Crude ash	4.91	Tyrosine	11.40				
Crude fiber	6.72	Phenylalanine	5.30				
Protein Digestibility	85.06	Histidine	4.78				
		Lysine	7.12				

d.m., dry matter.
